# Complementary Roles of RT-PCR and Next-Generation Sequencing in Post-Vaccination SARS-CoV-2 Infection: Clinical Diagnosis, Genomic Characterization, and Surveillance

**DOI:** 10.3390/vaccines14090812

**Published:** 2026-09-15

**Authors:** Vasiliki E. Georgakopoulou, Vassiliki C. Pitiriga

**Affiliations:** 1 Department of Pathophysiology, Laiko General Hospital, National and Kapodistrian University of Athens, 11527 Athens, Greece; 2 Department of Microbiology, Medical School of Athens, National and Kapodistrian University of Athens, 75 Mikras Asias Street, 11527 Athens, Greece

**Keywords:** SARS-CoV-2, post-vaccination infection, breakthrough infection, RT-PCR, next-generation sequencing, genomic surveillance

## Abstract

SARS-CoV-2 infection after vaccination remains a clinical and public-health challenge because protection against infection may vary with time since vaccination, host factors, previous infection, and ongoing viral evolution. Although vaccination substantially reduces the risk of severe COVID-19, post-vaccination infections may still occur and may be mild, atypical, or asymptomatic. This narrative review examines the complementary roles of reverse-transcription polymerase chain reaction (RT-PCR) and next-generation sequencing (NGS) in the diagnosis, genomic characterization, and surveillance of post-vaccination SARS-CoV-2 infection. RT-PCR remains the first-line method for confirming acute infection because it is rapid, widely available, and clinically actionable, although its clinical performance depends on appropriate specimen collection and timing. NGS complements RT-PCR by providing viral genomic characterization, including lineage assignment, mutation detection, investigation of suspected transmission clusters, and population-level surveillance. Sequencing may provide additional value in selected settings, particularly suspected reinfection, persistent infection in selected immunocompromised patients, outbreak investigations, and representative or event-triggered genomic surveillance. Its use should therefore be guided by a predefined clinical, epidemiological, or surveillance objective rather than applied routinely to all post-vaccination infections. An integrated strategy combining first-line RT-PCR with objective-driven sequencing can preserve diagnostic efficiency while providing genomic information when it is most likely to influence patient-level investigation, infection-control assessment, or public-health surveillance.

## 1. Introduction

The widespread implementation of coronavirus disease 2019 (COVID-19) vaccination has substantially reduced the risk of severe acute respiratory syndrome coronavirus 2 (SARS-CoV-2)-related hospitalization, severe disease, and death. Nevertheless, SARS-CoV-2 infections continue to occur after vaccination because protection against infection is influenced by time since vaccination, previous infection, host immune status, exposure intensity, and ongoing viral evolution [1,2,3]. In this review, the neutral term “post-vaccination infection” is used preferentially, whereas “breakthrough infection” is retained when referring to studies or surveillance definitions that specifically employed this terminology.

Accurate laboratory testing remains essential for individual patient management, infection control, outbreak investigation, and epidemiological surveillance. Real-time reverse-transcription polymerase chain reaction (RT-PCR) remains the principal method for confirming acute SARS-CoV-2 infection because validated assays are rapid, widely available, and generally demonstrate high analytical sensitivity and specificity [4,5]. Clinical sensitivity, however, depends on factors such as specimen type, sampling quality, anatomical site, timing relative to exposure or symptom onset, and viral kinetics. These considerations are particularly relevant in vaccinated individuals, in whom infection may be mild, atypical, or asymptomatic and viral kinetics may vary according to host and viral factors [6].

Routine RT-PCR confirms the presence of SARS-CoV-2 RNA but provides limited genomic information. Although selected assays can identify predefined mutations or target-failure patterns, they cannot provide comprehensive lineage assignment, characterize unexpected genomic changes, or reliably assess genomic relatedness among cases during suspected outbreaks [7,8]. These limitations define the circumstances in which additional genomic characterization may be useful.

Next-generation sequencing (NGS) complements RT-PCR by enabling partial or near-complete characterization of the viral genome. Depending on sample quality, viral RNA quantity, sequencing approach, genome coverage, and bioinformatic workflow, NGS can support lineage assignment, mutation detection, investigation of suspected transmission clusters, longitudinal genomic comparison, and monitoring of viral evolution at the population level [9,10,11]. Genomic surveillance has played a central role in identifying and tracking major SARS-CoV-2 variants and their descendant lineages and remains important as viral evolution continues [2,12,13].

The respective roles of RT-PCR and NGS can be considered at three levels. At the individual diagnostic level, RT-PCR provides rapid confirmation of acute infection and supports immediate clinical and infection-control decisions. At the patient-specific genomic level, NGS may provide additional information in selected circumstances, including suspected reinfection, persistent infection in selected immunocompromised patients, unusual treatment-associated events, and clinically or epidemiologically important cases. At the population level, sequencing supports representative genomic surveillance, detection of emerging lineages, and assessment of evolutionary trends over time [11,14,15].

The central issue is therefore not whether RT-PCR or NGS is the superior molecular method, but how each should be used according to the clinical or public-health objective. RT-PCR and NGS should be viewed as complementary technologies with distinct functions, turnaround times, information outputs, and resource requirements. Sequencing is most informative when a specific downstream question has been defined, such as whether genomic data may clarify suspected reinfection, contribute to an outbreak investigation, characterize viral evolution in a selected immunocompromised patient, or support population-level surveillance.

Despite extensive literature on SARS-CoV-2 molecular diagnostics and genomic surveillance, the post-vaccination setting introduces additional interpretive complexity because vaccination history, booster exposure, previous infection, hybrid immunity, immune competence, viral kinetics, circulating lineages, and sequencing-selection strategies vary substantially across populations. A unified framework is therefore needed to distinguish acute diagnostic testing from patient-specific genomic characterization and population-level surveillance.

This narrative review critically examines the complementary roles of RT-PCR and NGS in post-vaccination SARS-CoV-2 infection. Particular emphasis is placed on their analytical and operational characteristics, the clinical and epidemiological circumstances in which sequencing may provide incremental information, and the development of an integrated, objective-driven framework for their use in clinical and public-health practice.

This narrative review was informed by a targeted literature search of PubMed/MEDLINE, Scopus, and Web of Science for publications available from January 2020 through 15 August 2026. The search combined terms related to SARS-CoV-2 and vaccination with terms addressing molecular diagnosis and genomic characterization, including combinations of “SARS-CoV-2” OR “COVID-19” with “post-vaccination infection” OR “breakthrough infection,” “RT-PCR” OR “real-time RT-PCR,” “cycle threshold” OR “Ct value,” “next-generation sequencing” OR “whole-genome sequencing,” “genomic surveillance,” “reinfection,” “persistent infection,” “outbreak investigation,” “vaccine effectiveness,” and “emerging diagnostic technologies.” Searches were limited to publications in English; no restriction was applied according to geographical setting. Peer-reviewed original studies, methodological studies, genomic-surveillance reports, relevant reviews, and guidance documents from recognized international public-health organizations were considered. Publications were included when they provided information directly relevant to the diagnostic performance or interpretation of RT-PCR, the technical or operational use of NGS, genomic surveillance, or the integration of these approaches in vaccinated populations. Publications without direct relevance to SARS-CoV-2 molecular diagnosis or genomic characterization, purely preclinical reports without implications for the review objectives, conference abstracts lacking sufficient methodological information, and duplicate publications were excluded. Additional sources were identified through screening the reference lists of included and highly relevant articles and were retained when they provided methodological, clinical, genomic, or public-health information directly relevant to the scope of the review. Both authors participated in the selection and evaluation of the literature, with disagreements resolved by discussion and consensus. Because the included literature was heterogeneous with respect to study design, vaccine exposure, circulating lineages, specimen selection, and sequencing workflows, no formal meta-analysis, risk-of-bias assessment, or certainty-of-evidence grading was performed.

## 2. SARS-CoV-2 Infection After Vaccination: Clinical and Virological Background

### 2.1. Definition of Post-Vaccination Infection

SARS-CoV-2 infection after vaccination has commonly been described in the earlier literature as “breakthrough infection.” Early surveillance definitions generally defined breakthrough infection as SARS-CoV-2 infection occurring at least 14 days after completion of a primary vaccination series [16,17]. However, this definition has become increasingly difficult to apply uniformly because contemporary populations have heterogeneous vaccination and booster histories, updated vaccine formulations, previous SARS-CoV-2 infection, and hybrid immunity. Accordingly, the term “post-vaccination infection” is used preferentially in this review, while “breakthrough infection” is retained when referring to studies or surveillance definitions that specifically employed this terminology.

The term breakthrough infection should not be interpreted as complete vaccine failure. COVID-19 vaccines were primarily developed and evaluated to reduce symptomatic infection, severe disease, hospitalization and mortality, rather than to guarantee absolute and durable sterilizing immunity in every vaccinated individual [18]. Therefore, the occurrence of SARS-CoV-2 infection after vaccination reflects the biological reality that vaccine-induced protection against infection is incomplete and may diminish over time, particularly when host vulnerability and viral evolution coexist. Importantly, the detection of infection after vaccination does not by itself indicate inadequate vaccine effectiveness at the individual or population level, because protection against severe outcomes may remain substantial even when protection against infection has declined.

### 2.2. Why Post-Vaccination Infections Occur

Post-vaccination infections occur because protection against SARS-CoV-2 infection is influenced by interacting host, vaccine, exposure and viral factors. One of the most important host-related mechanisms is waning immunity. Neutralizing antibody levels decline over time after vaccination, and lower peri-infection neutralizing antibody titres have been associated with a higher risk of post-vaccination infection [19]. In a cohort of vaccinated healthcare workers, Bergwerk et al. showed that individuals with breakthrough infection had lower neutralizing antibody titres than matched uninfected controls, supporting the role of humoral immunity in protection against infection [19]. Nevertheless, memory B-cell responses and T-cell immunity may continue to protect against severe disease even when protection against infection decreases. The relationship between these immune components and the risk of infection, however, is not uniform across vaccine platforms, booster schedules, viral lineages and patient populations.

Incomplete vaccine response is another important contributor. Older adults, patients with multiple comorbidities and immunocompromised individuals may develop weaker or less durable immune responses after vaccination [20]. This is particularly relevant for patients receiving B-cell-depleting therapies, transplant recipients, individuals with hematological malignancies and patients receiving intensive immunosuppression. In these groups, post-vaccination infection may reflect inadequate generation or persistence of protective immunity rather than escape from a fully developed immune response. Previous infection and hybrid immunity may further modify this risk, although the magnitude and durability of protection vary according to the interval since infection or vaccination, the infecting lineage and host characteristics.

Virus-related factors also play a central role. The emergence of variants with increased transmissibility or partial immune escape has altered the epidemiology of post-vaccination infection. The Delta variant was associated with increased transmissibility and high viral RNA burden in some vaccinated individuals, whereas Omicron and its sublineages demonstrated substantial antigenic divergence from earlier strains, reducing neutralizing antibody activity after prior infection or vaccination [6,14,21]. These variants do not abolish all vaccine protection, but they can reduce protection against infection and symptomatic disease, especially when antibody levels have declined. The clinical effect of such variants nevertheless depends on the interaction between viral characteristics and host immunity.

Exposure-related factors further modify the probability of post-vaccination infection. Healthcare workers, household contacts of infected individuals, residents of congregate settings and persons exposed during periods of intense community transmission may experience repeated or prolonged exposure. Repeated or intense exposure may further increase the probability of infection, although exposure dose is rarely quantified directly in clinical studies.

Post-vaccination infection is therefore best understood as the result of a dynamic interaction among time since vaccination, vaccine platform and booster status, previous infection, immune competence, exposure circumstances and the biological characteristics of the circulating lineage.

### 2.3. Clinical Presentation After Vaccination

The clinical presentation of SARS-CoV-2 infection after vaccination is highly variable. Many post-vaccination infections are asymptomatic or mildly symptomatic, especially among younger and immunocompetent individuals [16,19]. In early Centers for Disease Control and Prevention surveillance data, a substantial proportion of reported breakthrough infections were asymptomatic, although passive surveillance likely underestimated mild or untested infections [16]. Symptomatic cases may present with upper respiratory symptoms, fever, headache, myalgia, fatigue, cough, sore throat, anosmia or gastrointestinal complaints. Compared with infections in unvaccinated individuals, symptoms after vaccination are often attenuated, but this pattern is not universal and may differ across viral lineages, time since vaccination, previous infection and host immune status.

In the study by Bergwerk et al., most post-vaccination infections among vaccinated healthcare workers were mild or asymptomatic, although persistent symptoms were reported in some individuals [19]. Hacisuleyman et al. similarly described variant-associated infections in fully vaccinated individuals [2]. However, these early vaccine-era observations may not be directly generalizable to contemporary populations with different booster schedules, hybrid immunity, and post-Omicron lineages.

Severe post-vaccination disease is less common but clinically important. It occurs more frequently in older adults, individuals with underlying comorbidities and patients with impaired immune responses [20]. Severe disease after vaccination may also be associated with a long interval since the most recent vaccine dose or infection by a lineage with partial immune-evasive properties. Therefore, a history of vaccination should reduce, but not eliminate, concern for COVID-19 in patients with compatible respiratory illness, hypoxemia or systemic inflammatory features.

The altered clinical spectrum after vaccination has practical diagnostic consequences. Mild or atypical symptoms may delay testing, and asymptomatic infections may be detected only through screening, outbreak investigations or pre-procedural testing. In hospitals and long-term care facilities, this creates a need for diagnostic strategies that combine clinical assessment with exposure history, vaccination status, immune status, local epidemiology and molecular testing. Vaccination status should inform risk stratification but should not be used as a reason to exclude SARS-CoV-2 testing when clinical or epidemiological suspicion is present.

### 2.4. Post-Vaccination Viral Kinetics and Transmissibility

Vaccination may modify SARS-CoV-2 viral kinetics, but the magnitude and duration of detectable viral RNA after post-vaccination infection vary according to the infecting lineage, time since vaccination, previous infection, immune status, symptom timing, and sampling strategy. Consequently, findings from one vaccination or variant era should not be assumed to apply uniformly to later epidemiological settings.

During the Delta-predominant period, several studies raised concern that vaccinated individuals with post-vaccination infection could have substantial amounts of detectable viral RNA at the time of diagnosis. In an outbreak investigation in Barnstable County, Massachusetts, most sequenced specimens were identified as the Delta variant, and Ct values among vaccinated and unvaccinated infected individuals were broadly similar [21]. Chau et al. also reported low Ct values, consistent with a relatively high viral RNA burden, among vaccinated healthcare workers infected with the Delta variant in Vietnam, although most infections were asymptomatic or mild [6]. These findings showed that substantial viral RNA burden could occur after vaccination during Delta circulation, although single-time-point measurements could not define the subsequent duration of shedding or transmission risk.

The applicability of these early Delta-era findings to later vaccination schedules, hybrid immunity, and post-Omicron lineages is uncertain. Viral kinetics may differ according to the infecting lineage, time since the most recent vaccine dose, previous infection, immune status, symptom timing, and sampling strategy. Consequently, comparisons across studies should be interpreted cautiously, particularly when vaccination status, specimen collection, and timing of testing are not standardized.

## 3. RT-PCR in the Diagnosis of Post-Vaccination SARS-CoV-2 Infection

### 3.1. Principles of RT-PCR

RT-PCR remains the most widely used molecular method for the diagnosis of acute SARS-CoV-2 infection. The diagnostic process begins with appropriate specimen collection, most commonly from the upper respiratory tract using nasopharyngeal, nasal, or combined nasal–oropharyngeal swabs, although lower respiratory tract specimens may be used in selected hospitalized patients with pneumonia or high clinical suspicion despite negative upper respiratory tract testing [5,22]. Correct sampling technique is essential because inadequate specimen collection may reduce viral RNA recovery and increase the risk of false-negative results.

After collection, viral RNA is extracted from the clinical specimen and purified to remove inhibitors that may interfere with amplification. The RNA is then reverse transcribed into complementary deoxyribonucleic acid (cDNA), which serves as the template for polymerase chain reaction amplification. During amplification, primers and probes target specific conserved regions of the SARS-CoV-2 genome. Commonly used targets include the envelope gene, nucleocapsid gene, RNA-dependent RNA polymerase gene, open reading frame 1ab, and spike gene, depending on the assay platform and laboratory protocol [4,23]. Because mutations may affect primer- or probe-binding sites, multi-target assays are generally more robust than single-target designs, although no assay is completely unaffected by viral evolution.

Real-time RT-PCR detects the accumulation of amplified products during each amplification cycle through fluorescent signals. When viral genetic material is present in sufficient quantity, fluorescence crosses a predefined threshold, generating a positive result. The cycle at which this occurs is reported as the Ct value. In diagnostic practice, assays usually include internal controls to confirm specimen adequacy, extraction efficiency, and the absence of amplification inhibitors. These controls are important because molecular failure may otherwise be misclassified as a true negative result.

The original SARS-CoV-2 RT-PCR protocols were rapidly developed and validated early in the pandemic, allowing molecular diagnosis to be implemented internationally [4]. Since then, multiple commercial and laboratory-developed assays have been introduced, many using multiplex designs that target more than one viral genomic region. Multiplexing improves diagnostic robustness because detection does not depend on a single viral target, which is particularly relevant as SARS-CoV-2 continues to evolve. Nevertheless, differences in assay design, extraction procedures, amplification chemistry, positivity thresholds, and quality-control systems contribute to variability across laboratories and should be considered when comparing results.

### 3.2. Diagnostic Performance

RT-PCR is considered the reference laboratory method for the confirmation of acute SARS-CoV-2 infection in most clinical settings because properly validated assays generally demonstrate high analytical sensitivity and specificity [4,5]. Analytical sensitivity refers to the ability of an assay to detect a defined amount of viral RNA under controlled conditions, whereas clinical sensitivity reflects the probability of detecting infection in an individual patient. These concepts should not be used interchangeably. Properly validated RT-PCR assays generally provide high analytical specificity through the use of SARS-CoV-2-specific primer and probe targets [4].

Diagnostic performance, however, is not determined by analytical sensitivity alone. The probability of detecting SARS-CoV-2 RNA depends strongly on the timing of testing in relation to exposure and symptom onset. Kucirka et al. showed that the false-negative rate of RT-PCR varies substantially across the course of infection, being higher very early after exposure and increasing again later as detectable viral RNA declines [24]. Therefore, a negative RT-PCR result cannot always exclude infection when testing is performed outside the optimal diagnostic window or when clinical suspicion remains high. Clinical sensitivity is also influenced by specimen type, anatomical sampling site, sampling technique, specimen transport, viral kinetics, and the presence of lower respiratory tract disease.

In vaccinated individuals, clinical sensitivity may also be influenced by altered viral kinetics, which vary across viral lineages, vaccination and booster histories, previous infection, immune status, and sampling strategies [6,21]. These factors should therefore be considered when interpreting RT-PCR results in the post-vaccination setting.

When an initial RT-PCR result is negative, but clinical or epidemiological suspicion remains substantial, repeat testing may be appropriate, particularly when the first specimen was obtained early after exposure or symptom onset, specimen adequacy is uncertain, or symptoms are evolving. Alternative specimen types, including lower respiratory tract samples in selected hospitalized patients, may also be considered according to the clinical presentation.

RT-PCR therefore remains the first-line molecular method for confirmation of acute SARS-CoV-2 infection, while genomic characterization is reserved for defined clinical, epidemiological, or surveillance objectives.

### 3.3. Interpretation of Cycle-Threshold Values

The Ct value represents the amplification cycle at which the fluorescent signal of a real-time RT-PCR assay exceeds the predefined detection threshold. In general, lower Ct values correspond to a greater amount of detectable viral RNA in the tested specimen, whereas higher Ct values indicate a smaller amount of detectable RNA [7]. This inverse relationship may provide contextual information on the relative amount of detectable viral RNA when interpreted together with symptom timing, specimen type, sampling quality, and the specific assay used.

Ct values should not be interpreted as standardized measures of viral load unless the assay has been specifically calibrated for quantitative reporting. They vary according to assay platform, primer–probe design, extraction method, amplification efficiency, specimen type, sampling quality, and other pre-analytical factors [7,25]. Consequently, Ct values are not directly interchangeable across assays or laboratories.

Ct values should not be used alone to infer infectivity. RT-PCR detects viral RNA rather than replication-competent virus, and both low and high Ct values require interpretation in relation to the stage of infection, specimen quality, symptoms, and host factors. A low Ct value may indicate a greater amount of detectable viral RNA, whereas a high Ct value may reflect early infection, resolving infection, residual RNA detection, poor sampling, or genuinely low RNA concentration [7].

There is no universal Ct threshold for infectivity, clinical severity, release from isolation, or sequencing eligibility. When Ct values are used to support specimen selection for sequencing, thresholds should be assay-specific, locally validated, and interpreted together with specimen quality and the purpose of sequencing. Ct values should support, rather than replace, clinical and epidemiological assessment.

### 3.4. Advantages of RT-PCR

RT-PCR has several advantages that explain its continued role as the primary molecular diagnostic method for SARS-CoV-2 infection after vaccination. First, it provides rapid results compared with most sequencing workflows. Many clinical laboratories can deliver RT-PCR results within hours, making the method suitable for acute clinical decision-making, patient isolation, hospital admission pathways, and occupational health screening. Its principal advantage is therefore not only analytical performance but also the ability to generate clinically actionable results within a relevant decision-making timeframe.

Second, RT-PCR is widely available and supported by extensive laboratory infrastructure. During the pandemic, molecular testing capacity expanded rapidly, allowing RT-PCR to be implemented in hospitals, public-health laboratories, and private diagnostic centres. Standardized protocols, commercial kits, internal controls, and external quality-assurance procedures have improved reproducibility and made RT-PCR suitable for high-throughput testing [4,5].

Third, RT-PCR is generally more cost-effective than NGS for routine diagnosis. Although costs vary between countries, laboratory systems, assay volumes, and procurement models, RT-PCR usually requires less complex infrastructure, shorter processing time, and less bioinformatic support than sequencing. This makes it more feasible for routine diagnosis, repeat testing, and large-scale screening. Cost differences nevertheless vary according to laboratory scale, workflow, and local setting.

Fourth, RT-PCR is flexible. Multiplex assays can detect several SARS-CoV-2 targets simultaneously, reducing the risk of diagnostic failure caused by mutation in a single target region. Some platforms can also include mutation-specific targets or target-failure patterns that provide rapid presumptive information about selected variants. Such approaches may be useful for screening when the relevant mutations are already known and epidemiologically informative, but they cannot characterize unexpected mutations or replace genome sequencing for definitive lineage assignment.

These characteristics maintain the central role of RT-PCR in routine diagnosis of post-vaccination SARS-CoV-2 infection.

### 3.5. Limitations of RT-PCR in Vaccinated Populations

Despite its strengths, RT-PCR has important limitations in the diagnosis and interpretation of SARS-CoV-2 infection after vaccination. The first limitation is the possibility of false-negative results. False negatives may occur when testing is performed too early after exposure, when the amount of viral RNA is below the assay’s limit of detection, when sampling is inadequate, when specimen transport or storage is suboptimal, or when infection is predominantly located at an anatomical site not adequately represented by the collected specimen [24]. In vaccinated individuals, modified viral kinetics may narrow the period of optimal detection in some circumstances, although this effect is heterogeneous and should not be assumed to occur uniformly.

A second limitation is that routine RT-PCR does not fully characterize the infecting virus. Most diagnostic assays confirm the presence of SARS-CoV-2 RNA but do not determine the complete viral genome or assign a lineage. This limitation is most relevant when the objective extends beyond infection confirmation to genomic surveillance, assessment of suspected reinfection, investigation of an outbreak, or evaluation of unusual treatment-associated viral evolution. Mutation-specific RT-PCR can detect selected known mutations, but it cannot identify unexpected mutations, novel combinations of mutations, or newly emerging lineages outside its predefined targets.

A third limitation is the restricted ability of RT-PCR to investigate transmission chains. Although RT-PCR can confirm infection in multiple individuals, it does not provide sufficient genomic resolution to distinguish epidemiologically linked cases from separate viral introductions.

A fourth limitation is dependence on known genomic targets. RT-PCR assays require primer and probe binding to specific viral sequences. Although most assays target conserved regions and many use multiple targets, viral mutations may occasionally reduce assay sensitivity or cause failure of a specific target. This phenomenon was observed with spike-gene target failure in some assays during the emergence of the Alpha variant, where it became useful as a screening marker but also illustrated how viral evolution can affect molecular test performance [8]. Multi-target assay design reduces, but does not completely eliminate, this risk.

Finally, RT-PCR detects viral RNA rather than replication-competent virus and therefore cannot independently determine infectiousness.

## 4. Next-Generation Sequencing in SARS-CoV-2 Diagnosis and Surveillance

### 4.1. Principles and Main Analytical Approaches

NGS refers to a group of high-throughput molecular technologies that allow the simultaneous sequencing of large numbers of nucleic acid fragments. In the context of SARS-CoV-2, NGS can be used to generate partial, near-complete, or complete viral genome sequences from clinical specimens. Unlike routine RT-PCR, which detects predefined viral targets, sequencing provides broader genomic information and can identify mutations across adequately covered regions of the viral genome [10,26]. NGS should not, however, be considered a uniform analytical method. Its performance and interpretability depend on the enrichment strategy, sequencing platform, read depth, genome coverage, consensus-calling criteria, and bioinformatic workflow used.

Whole-genome sequencing aims to recover the complete or near-complete SARS-CoV-2 genome. This approach is particularly useful for lineage assignment, phylogenetic analysis, longitudinal genomic comparison, and monitoring viral evolution. In most workflows, viral RNA is reverse-transcribed, amplified or enriched, sequenced, and processed through bioinformatic pipelines for quality control, genome assembly, mutation calling, and lineage assignment [10,26]. Incomplete genome recovery is common when viral RNA quantity or quality is insufficient, and the term “whole-genome sequencing” should not imply that every nucleotide position is reliably determined in every specimen.

Targeted amplicon sequencing is one of the most widely used approaches for SARS-CoV-2 genomic surveillance. In this method, sets of primers amplify overlapping regions of the viral genome before sequencing. Amplicon-based methods are relatively sensitive, scalable, and cost-effective compared with unbiased metagenomic approaches, making them suitable for large surveillance programmes [26,27].

Amplicon-based sequencing nevertheless has important technical limitations. Mutations in primer-binding sites may cause reduced amplification, uneven coverage, or complete amplicon dropout. Amplification artefacts, contamination, and preferential amplification may also affect consensus calling. These limitations are particularly relevant when new lineages emerge with mutations in regions targeted by established primer schemes. Protocols and primer sets therefore require continued evaluation and periodic updating.

Metagenomic sequencing is a less targeted approach in which all nucleic acids present in a specimen may be sequenced. This strategy can detect SARS-CoV-2 without relying on virus-specific amplification and may also identify co-infecting respiratory pathogens. However, metagenomic sequencing is generally more expensive, analytically complex, and less sensitive when SARS-CoV-2 RNA abundance is low because human and bacterial nucleic acids may dominate the specimen [10,26]. For this reason, metagenomic sequencing is valuable in selected research, pathogen discovery, or complex diagnostic contexts, whereas targeted amplicon sequencing remains more practical for routine SARS-CoV-2 surveillance.

Hybrid-capture enrichment represents an intermediate strategy in which viral nucleic acid is enriched using complementary probes before sequencing. It may provide more even genomic coverage and be less vulnerable than amplicon sequencing to individual primer mismatches, but it is generally more expensive, requires additional laboratory steps, and may remain less sensitive than targeted amplification when viral RNA abundance is low.

Commonly used sequencing platforms also differ in ways that influence workflow and interpretation. Illumina and Oxford Nanopore platforms differ in read characteristics, accuracy, speed, workflow flexibility, and equipment requirements; these platform-specific features should be considered when comparing sequencing results across laboratories.

A further distinction is required between consensus-level sequencing and minority-variant analysis. Routine surveillance sequencing usually reports a consensus genome representing the dominant viral population in the specimen. It may not reliably identify low-frequency within-host variants. Detection and interpretation of minority variants require validated sequencing depth, allele-frequency thresholds, strand-balance criteria, contamination controls, and, where appropriate, technical replication. This distinction is particularly important in immunocompromised patients with prolonged infection, in whom within-host evolution may occur.

### 4.2. Mutation Detection and Lineage Assignment

NGS identifies SARS-CoV-2 genomic changes by detecting nucleotide substitutions, deletions, insertions, and other differences relative to a reference sequence. These changes can then be interpreted in relation to recognized viral lineages and variants under monitoring. Mutations may affect viral antigenicity, replication, diagnostic assay performance, or susceptibility to targeted therapies, depending on their genomic location and biological effect [28,29].

Mutation detection should be distinguished from demonstration of a biological or clinical phenotype. Genomic findings require interpretation in conjunction with experimental, clinical, immunological, and epidemiological evidence.

Lineage assignment is usually performed using dynamic nomenclature systems and bioinformatic tools. Lineage assignment is commonly performed using dynamic nomenclature systems such as Pango and associated bioinformatic tools such as Pangolin, which classify SARS-CoV-2 sequences according to their evolutionary relationships [30,31].

Lineage assignment is not completely static or platform-independent. Classification systems, reference databases, and software versions are updated as viral diversity increases. The same sequence may therefore receive a revised lineage designation after database or algorithm updates. Sequencing studies should report the software, database, and version used, together with genome-quality and coverage criteria.

Variant identification through NGS is broader than mutation-specific RT-PCR screening because sequencing can detect known and emerging mutations across adequately covered genomic regions [14,28,29]. However, incomplete genomic coverage may result in uncertain or incorrect lineage assignment.

The distinction between mutation detection and lineage assignment is clinically and epidemiologically important because lineage assignment places individual genomic findings within a broader evolutionary framework. This facilitates monitoring of lineage frequency and viral evolution, while phenotype attribution requires additional functional and epidemiological evidence.

### 4.3. Role in Genomic Surveillance

Genomic surveillance is one of the most important applications of NGS in the COVID-19 era. At the population level, sequencing allows systematic monitoring of circulating SARS-CoV-2 lineages, detection of emerging variants, and assessment of viral evolution over time. The World Health Organization has emphasized that genomic sequencing should be implemented to maximize public-health impact, including early identification of variants, monitoring of viral spread, and support for control measures [26]. Similarly, the European Centre for Disease Prevention and Control has recommended combining representative and targeted sequencing strategies for SARS-CoV-2 monitoring [11].

Representative genomic surveillance estimates the distribution of viral lineages within a defined population. This approach can reveal whether a lineage is increasing in frequency, replacing previously circulating lineages, or spreading across regions. It also allows comparison between time periods, geographical areas, and population groups. Such surveillance was crucial for tracking the emergence and expansion of Alpha, Delta, and Omicron lineages [12,13,14,32].

For representative surveillance to generate valid estimates, sample selection should be independent of unusual clinical severity, host risk, or Ct value whenever feasible. Preferential sequencing of severe cases, immunocompromised patients, treatment failures, or low-Ct specimens may be valuable for targeted investigation but can distort estimates of lineage frequency, disease severity, or apparent vaccine escape. Representative and targeted surveillance should therefore be analyzed and reported separately.

Targeted genomic surveillance focuses on specimens with particular clinical or epidemiological relevance. These may include unusual clusters, suspected reinfections, infections in selected immunocompromised patients, unexpected diagnostic target failures, travel-associated introductions, treatment-associated events, or outbreaks in healthcare facilities. Severe post-vaccination disease may warrant sequencing when it occurs within an unusual clinical or epidemiological pattern.

When sequencing capacity is limited, prioritization may follow four complementary categories: representative sampling for unbiased population surveillance; event-triggered sequencing for unusual clinical or epidemiological events; rapid sequencing during active outbreaks when results may still influence control measures; and longitudinal sequencing in selected immunocompromised patients with evidence suggesting persistent viral replication.

Genomic surveillance can inform risk assessment, infection-control responses, vaccine-strain updates, and international data sharing. Its interpretation should be integrated with relevant clinical and epidemiological information.

### 4.4. Role in Outbreak Investigation

NGS can support outbreak investigation by comparing viral genomes from cases that may be epidemiologically linked. In settings such as hospitals, long-term care facilities, schools, universities, and workplaces, multiple SARS-CoV-2 cases may occur within a short period. Conventional epidemiology can identify possible links through time, place, and contact history, but it may not distinguish a single transmission cluster from multiple community introductions.

Sequencing adds resolution by comparing consensus genomes from different cases. When sequences are identical or closely related and cases occur within a compatible epidemiological timeframe, this may support the hypothesis of linked transmission. Conversely, when sequences belong to clearly different lineages or show genomic differences inconsistent with the suspected timeframe, a common outbreak source becomes less likely.

Genomic similarity can strengthen or weaken an outbreak hypothesis but cannot independently establish direct transmission or its direction. Sequencing findings should therefore be interpreted together with temporal, spatial, and contact-tracing data and the background diversity of circulating viruses.

This approach has been applied in healthcare and institutional settings. Genomic epidemiology studies have shown that integrating sequencing with infection-prevention data can help distinguish probable hospital-associated transmission from multiple community introductions and can clarify transmission patterns during suspected outbreaks [33]. In university or other congregate settings, sequencing has also been used to assess whether apparent clusters reflect one dominant transmission chain or multiple introductions [34].

In post-vaccination outbreaks, sequencing can determine whether cases belong to the same lineage and identify genomic features of potential epidemiological or antigenic relevance. These findings should be interpreted in the context of vaccination timing, population susceptibility, exposure conditions, infection-control practices, and background community transmission.

Sequencing results may support targeted control measures, such as enhanced testing, cohorting, contact investigation, environmental assessment, or review of infection-prevention procedures. Their practical value is greatest when the turnaround time is sufficiently short for the findings to influence an ongoing response. Results generated after an outbreak has ended may remain epidemiologically informative but have limited immediate operational impact.

### 4.5. Advantages and Incremental Value of NGS

The principal advantage of NGS is its ability to provide broad genomic characterization. By sequencing large portions or the entirety of the SARS-CoV-2 genome, NGS can support lineage assignment, define consensus mutation profiles, identify newly emerging genomic changes, and contribute to phylogenetic analysis [10,26]. This capability has been essential for the identification and monitoring of Alpha, Delta, Omicron, and subsequent lineages with altered antigenic or epidemiological characteristics [12,13,14,32].

NGS may provide incremental information in selected clinically or epidemiologically unusual situations, including suspected reinfection, persistent infection with evidence of ongoing replication, unusual treatment-associated events, and selected infections in immunocompromised patients. In immunocompromised hosts, prolonged replication may permit within-host viral evolution, making longitudinal sequencing useful for detecting changes that emerge over time under immune or therapeutic pressure [35].

Persistent infection should not be inferred from prolonged RT-PCR positivity alone. Longitudinal sequencing is most informative when persistent RNA detection is accompanied by compatible clinical or virological evidence, such as evolving viral RNA burden, culture positivity where available, or sequential genomic change.

Suspected reinfection should be assessed using appropriately separated infection episodes together with clinical and epidemiological information, intervening testing, and, when available, genomic differences between episodes. Sequencing may support but does not always resolve this distinction, particularly when the earlier specimen is unavailable.

Sequencing may also be useful in unusual treatment-associated events when viral evolution or reduced antiviral susceptibility is suspected. Interpretation requires appropriate temporal sampling and evidence linking the detected genomic change to altered therapeutic susceptibility.

NGS can also identify genomic changes that may affect molecular assay performance and thereby support investigation of unusual target-failure patterns or assay redesign.

### 4.6. Limitations of NGS

Despite its advantages, NGS has important limitations that restrict its use as a routine first-line diagnostic test. First, sequencing is generally more expensive and technically demanding than RT-PCR. It requires specialized equipment, sequencing reagents, library-preparation workflows, quality-control procedures, trained personnel, and computational support. These requirements may be difficult to sustain in resource-limited settings or laboratories without established molecular-surveillance infrastructure [10,26].

Second, NGS generally has a longer and more workflow-dependent turnaround time than routine RT-PCR because sequencing requires library preparation, sequence generation, quality control, bioinformatic analysis, and reporting. Rapid small-batch workflows may provide results within a clinically relevant timeframe, whereas centralized or batched surveillance systems may require several days.

Third, sequencing success depends on viral RNA quantity and quality, specimen collection, storage, and the sequencing workflow. Not every RT-PCR-positive specimen is suitable for sequencing, and no universal Ct cutoff guarantees genome recovery. Laboratories should therefore use locally validated, assay-specific specimen-selection criteria.

There is no universal Ct cutoff that guarantees sequencing success. The relationship between Ct and genome completeness varies by assay, specimen type, extraction method, sequencing protocol, and required quality threshold. Laboratories should establish locally validated, assay-specific specimen-selection criteria rather than applying a single universal Ct rule.

Fourth, amplicon-based sequencing is vulnerable to primer mismatch, uneven coverage, and amplicon dropout. Contamination, index misassignment, cross-sample carryover, amplification artefacts, and platform-specific errors may also affect mutation calling and lineage assignment. These risks require negative controls, appropriate indexing strategies, run-level quality control, contamination assessment, and validated criteria for accepting or rejecting a sequence.

Fifth, NGS requires validated bioinformatic workflows, updated reference databases, version control, and reproducible quality criteria. Differences in processing parameters and lineage-assignment software can produce discordant results; studies should therefore report software versions, relevant thresholds, and genome-quality criteria.

Sixth, routine consensus sequencing has limited sensitivity for minority variants. A consensus genome generally reflects the dominant viral population and may not detect low-frequency variants within a specimen. Claims regarding within-host evolution or emerging resistance require deep-sequencing methods with validated frequency thresholds and careful exclusion of sequencing error and contamination.

Finally, global access to sequencing remains unequal. Differences in laboratory capacity, sample coverage, workforce, data sharing, computational infrastructure, and integration with public-health systems can create geographical blind spots in variant surveillance [36]. Underrepresentation of low- and middle-income settings may delay the recognition of emerging lineages and reduce the global representativeness of sequence databases.

Feasible strategies in settings with limited resources include multi-target RT-PCR, sentinel representative sampling, event-triggered sequencing, regional sequencing hubs, shared bioinformatic infrastructure, and targeted mutation screening when routine whole-genome sequencing is not available. These approaches cannot fully replace representative NGS surveillance but may improve early detection and regional coverage.

## 5. Comparative and Complementary Roles of RT-PCR and NGS

RT-PCR and NGS have complementary roles in post-vaccination SARS-CoV-2 infection. RT-PCR provides rapid individual-level confirmation of acute infection, whereas NGS provides additional genomic information for selected patient-specific investigations and population-level surveillance. Their relative value therefore depends on the question being addressed rather than on direct analytical competition between the two methods.

### 5.1. Speed, Turnaround Time and Clinical Utility

The incremental value of NGS is greatest when genomic information addresses a predefined question that cannot be resolved by RT-PCR alone, such as suspected reinfection, longitudinal assessment of selected persistent infections, investigation of unusual treatment-associated events, or outbreak and surveillance objectives [26,35]. RT-PCR therefore has the greatest immediate diagnostic utility, whereas the incremental value of NGS is primarily genomic and epidemiological. For each sequencing request, the expected downstream use of the result should be defined in advance, such as outbreak-control assessment, reinfection evaluation, longitudinal genomic comparison, or contribution to genomic surveillance.

### 5.2. Analytical Sensitivity, Clinical Sensitivity and Sample Requirements

RT-PCR and NGS also differ in specimen requirements. Validated RT-PCR assays can detect relatively small amounts of viral RNA, whereas successful sequencing generally requires sufficient RNA quantity and quality to achieve adequate genome coverage. Ct values may assist local specimen-selection workflows but are assay-dependent and should not be used as universal sequencing thresholds. Accordingly, sequencing requires two separate considerations: whether a defined clinical, epidemiological, or surveillance objective exists and whether the specimen meets locally validated technical-quality criteria [37,38,39,40,41,42,43,44,45,46].

Preferential sequencing of only low-Ct or otherwise technically favourable specimens may introduce selection bias because infections with lower RNA burden, delayed presentation or different viral kinetics may be underrepresented. Such bias can distort estimates of lineage prevalence, disease severity or apparent vaccine escape. Representative surveillance and targeted sequencing should therefore be analyzed and reported separately [47].

### 5.3. Mutation Screening, Variant Identification and Lineage Assignment

Mutation-specific or target-failure RT-PCR assays can provide rapid presumptive information on predefined genomic changes, whereas NGS enables broader mutation detection and lineage assignment across adequately covered genomic regions [8,30,31]. The former may therefore support rapid screening of known targets, while the latter is better suited to characterization of emerging or unexpected genomic patterns.

### 5.4. Cost, Infrastructure and Accessibility

RT-PCR is generally more accessible, less resource-intensive, and more suitable for high-throughput routine diagnosis than NGS. Sequencing requires additional laboratory, computational, quality-control, and personnel infrastructure, and its cost and accessibility vary substantially across settings [4,5,10,26].

These resource differences support a model in which RT-PCR remains broadly available for first-line diagnosis, while NGS is deployed through representative, targeted, or event-triggered sequencing strategies according to local capacity and surveillance objectives [11,47].

### 5.5. Individual Diagnostic, Patient-Specific and Epidemiological Value

At the individual diagnostic level, RT-PCR provides rapid confirmation of detectable SARS-CoV-2 RNA and supports immediate clinical and infection-control assessment.

At the patient-specific level, NGS can provide additional genomic characterization in selected circumstances, including suspected reinfection, longitudinal investigation of persistent infection in selected immunocompromised patients, unusual treatment-associated events, and investigation of molecular target-failure patterns.

At the population level, NGS supports representative genomic surveillance, detection of emerging lineages, monitoring of lineage replacement and geographical spread, and genomic investigation of institutional outbreaks.

An integrated strategy should combine the strengths of both methods rather than use RT-PCR positivity as an automatic trigger for sequencing. Following first-line diagnostic testing, sequencing should be considered only when a defined clinical, epidemiological, or surveillance objective exists and the specimen meets locally validated technical-quality criteria.

The expected downstream use of the genomic result should be specified in advance, for example, outbreak-control assessment, reinfection evaluation, longitudinal genomic comparison, or surveillance reporting. This objective-driven approach distinguishes immediate diagnostic utility from patient-specific genomic characterization and population-level surveillance.

Table 1 illustrates the complementary characteristics of RT-PCR and NGS, highlighting their differences in purpose, analytical requirements, turnaround time, sample constraints, genomic information yield, clinical actionability, public-health utility, cost, infrastructure needs, and principal limitations.

**Figure 1:**
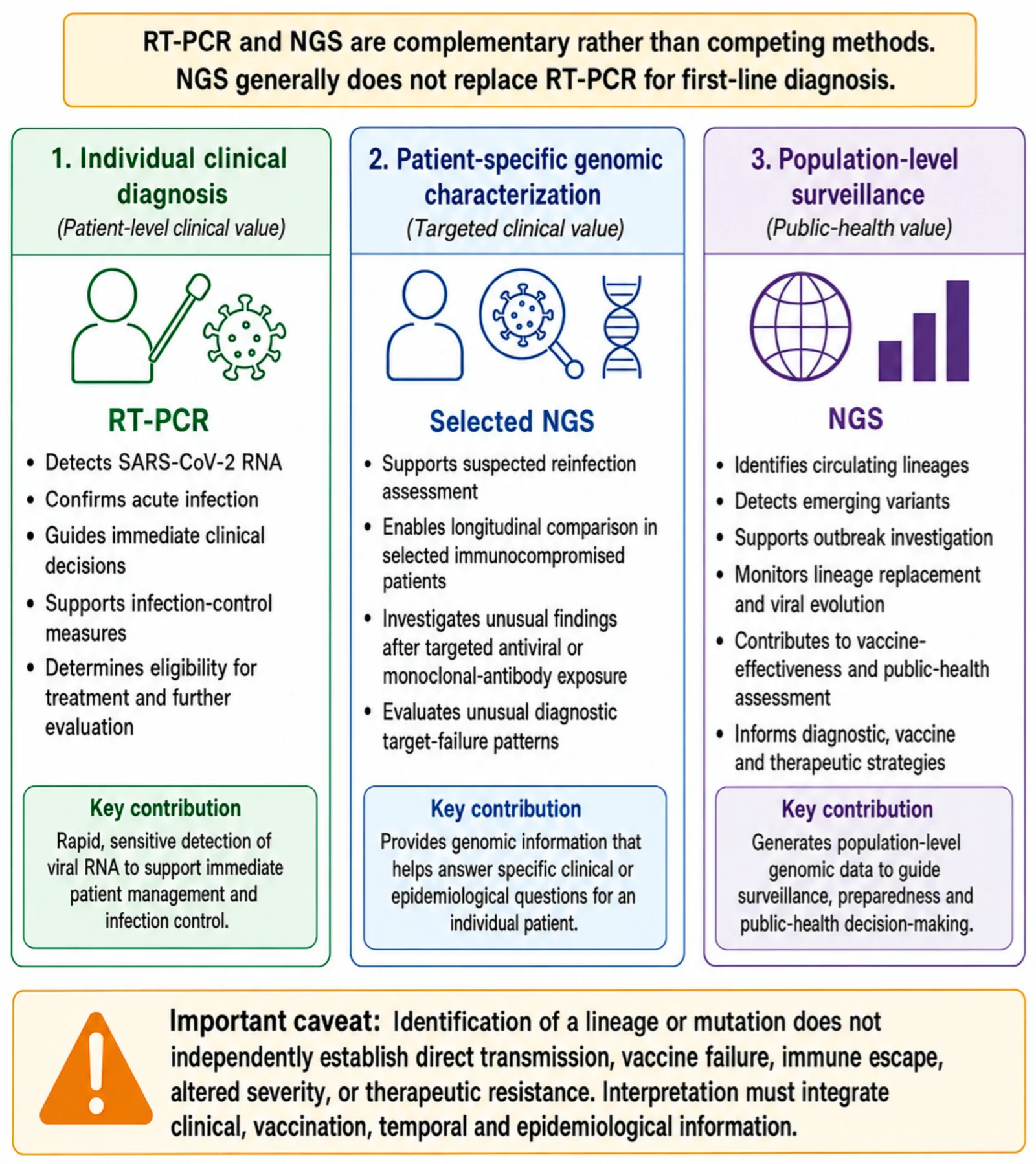
Complementary roles of RT-PCR and next-generation sequencing in post-vaccination SARS-CoV-2 infection.

The complementary diagnostic and public-health roles of RT-PCR and NGS are illustrated in Figure 1.

RT-PCR primarily provides rapid individual-level confirmation of infection and supports immediate clinical and infection-control decisions. NGS provides additional value in selected patient-specific investigations and in population-level genomic surveillance. Genomic findings require integration with clinical, epidemiological, and, where relevant, functional evidence and should not be interpreted in isolation as establishing transmission, vaccine failure, immune escape, altered severity, or therapeutic resistance.

## 6. Public Health Applications

### 6.1. Monitoring Vaccine Effectiveness

Genomic sequencing of SARS-CoV-2 infections occurring after vaccination has important public-health value because it can help identify lineages associated with changes in vaccine performance. Vaccine effectiveness is not a fixed property; it varies according to time since vaccination, host characteristics, vaccine platform, booster status, previous infection, hybrid immunity, and the antigenic characteristics of circulating lineages. Surveillance of post-vaccination infections should therefore not be limited to counting cases but should integrate genomic, clinical, epidemiological, and vaccination data whenever feasible.

Sequencing can identify whether increases in post-vaccination infections coincide with the expansion of particular lineages or mutation profiles, including lineages with antigenic changes [14,28]. However, lineage-specific effects on vaccine effectiveness require comparison with the source population and adjustment for relevant demographic, immunological, epidemiological, and temporal factors.

However, the detection of a lineage among vaccinated infected individuals does not by itself demonstrate reduced vaccine effectiveness. A valid assessment requires comparison with the distribution of the same lineage in the source population and adjustment for age, time since vaccination, booster exposure, previous infection, immune status, exposure risk, testing behaviour, calendar time, and geographical variation.

Linking vaccination status with genomic data may identify lineages disproportionately represented among post-vaccination infections. Such analyses remain vulnerable to differences in exposure, testing, healthcare-seeking behaviour, and sequencing success; preferential sequencing of severe or technically favourable specimens may introduce additional selection bias [47].

When genomic data are linked with appropriately designed epidemiological studies, they may support early recognition of lineage-associated reductions in protection against infection, symptomatic disease, hospitalization, or severe outcomes. The outcome under evaluation should be specified because a lineage may reduce protection against infection without producing an equivalent reduction in protection against severe disease.

NGS contributes to vaccine-effectiveness monitoring by providing genomic context to epidemiological observations. Quantification of lineage-specific effects nevertheless requires appropriately designed epidemiological studies together with complementary immunological or functional evidence.

### 6.2. Early Detection and Assessment of Emerging Variants

One of the most important public-health applications of NGS is the early detection of emerging SARS-CoV-2 lineages. SARS-CoV-2 continues to evolve through the accumulation of substitutions, deletions, insertions, and recombination events. Some lineages may acquire properties associated with increased transmissibility, altered antigenicity, diagnostic target failure, or partial immune escape. Genomic surveillance may allow these lineages to be recognized before they become dominant, creating an opportunity for earlier risk assessment and public-health response [26,48].

The emergence and expansion of Alpha, Delta, Omicron, and subsequent descendant lineages demonstrated that viral evolution could alter transmission dynamics, diagnostic strategies, vaccine policy, and clinical risk assessment [12,13,32]. These examples highlight why genomic surveillance remains relevant after widespread vaccination and prior infection.

Because sequencing is not restricted to predefined mutation targets, it can detect unexpected genomic changes and emerging lineages within adequately covered regions.

Identification of an emerging lineage should be followed by a broader assessment of its growth, geographical spread, antigenic characteristics, disease severity, diagnostic performance, therapeutic susceptibility, and vaccine effectiveness before clinical or public-health significance is assigned.

Once a lineage has been identified and characterized, targeted RT-PCR assays may provide rapid and economical screening, while sequencing remains necessary to monitor broader genomic evolution and the continued validity of screening targets.

The sensitivity of early-warning systems depends strongly on sampling design. Representative surveillance is required to estimate lineage frequencies, whereas targeted sequencing of unusual events may improve the probability of detecting rare variants. The two approaches should be used together because targeted sequencing alone cannot provide unbiased estimates of population prevalence [11,47,48].

### 6.3. Informing Vaccine-Antigen Updates and Vaccination Policy

Variant surveillance can contribute to decisions regarding vaccine-antigen composition and vaccination strategies. As SARS-CoV-2 evolves, the antigenic relationship between vaccine strains and circulating lineages may change. Genomic data may provide an early indication that a new lineage is expanding, whereas antigenic characterization, neutralization studies, immunogenicity data, epidemiological analyses, and clinical vaccine-effectiveness estimates determine whether that expansion is likely to affect vaccine performance [14,28,49].

The World Health Organization Technical Advisory Group on COVID-19 Vaccine Composition integrates genomic, antigenic, immunological, and vaccine-effectiveness evidence when advising on future vaccine-antigen composition [49].

Sequencing of post-vaccination infections may contribute to vaccine-strain assessment by identifying circulating lineages in populations with vaccine-induced or hybrid immunity. Decisions on vaccine updates, however, require integration of genomic findings with antigenic, immunological, epidemiological, and real-world vaccine-effectiveness data.

Variant surveillance may also inform population-level vaccination strategies when antigenically distinct lineages expand. Individual booster recommendations, however, are based on broader population-level evidence and national or international guidance rather than the sequence obtained from a single patient.

### 6.4. Global Surveillance Networks and Data Sharing

The public-health value of SARS-CoV-2 sequencing depends on timely data sharing and international collaboration. Viruses cross national borders, and lineages may emerge in one geographical region before spreading internationally. Timely sharing of genomic sequences, sampling dates, geographical information, and relevant epidemiological metadata is therefore essential for early detection, risk assessment, and coordinated response.

Platforms such as GISAID have supported international sharing of SARS-CoV-2 sequences and associated metadata, facilitating real-time monitoring of genomic diversity and lineage spread [50].

The value of a shared sequence depends on the quality and completeness of its associated metadata. Missing or inconsistent information on sampling date, geographical location, vaccination status, previous infection, age, clinical severity, immune status, and sequencing method may substantially limit epidemiological interpretation. At the same time, data sharing must comply with ethical, legal, privacy, and data-governance requirements.

Global genomic surveillance remains uneven. Sequencing capacity, sampling representativeness, turnaround time, data quality, and metadata completeness vary substantially across countries and regions, creating surveillance gaps that may delay recognition of emerging lineages [36,47]. Sequence counts alone should not be interpreted as equivalent to surveillance quality, because a large number of convenience samples may still provide a biased representation of viral circulation.

Unequal surveillance coverage may lead lineages to be first reported from countries with stronger sequencing systems, even when they did not originate there. The location of first detection should therefore not be assumed to represent the location of emergence.

Strengthening global surveillance requires more than distributing sequencing instruments. Sustainable systems also require trained personnel, stable reagent supply, quality-assurance programmes, bioinformatic infrastructure, secure data storage, rapid reporting pathways, and integration with clinical and epidemiological surveillance. Regional sequencing hubs, sentinel sampling, event-triggered investigation, and shared bioinformatic resources may improve coverage in settings where comprehensive national sequencing is not feasible [47,48].

A resilient global system should combine representative routine surveillance with targeted investigation of unusual clinical or epidemiological events. This model may improve both the unbiased monitoring of circulating lineages and the rapid detection of variants with potential public-health relevance.

## 7. Diagnostic Challenges in the Post-Vaccination Era

### 7.1. Mild or Atypical Clinical Presentation

Mild, atypical, or asymptomatic post-vaccination infection may delay recognition and testing, particularly when testing is not prompted by exposure, screening, hospital admission, or outbreak investigation.

This challenge is particularly relevant in healthcare and long-term care settings, where even mild infection may have consequences for vulnerable contacts. Vaccination status should therefore inform risk assessment without excluding testing when clinical or epidemiological suspicion is present.

A broader respiratory diagnostic approach may also be required when symptoms are non-specific, particularly during periods of simultaneous circulation of SARS-CoV-2, influenza viruses, respiratory syncytial virus, and other respiratory pathogens. In such settings, multiplex molecular testing may be more informative than SARS-CoV-2 testing alone.

### 7.2. Variable Viral Kinetics and Diagnostic Timing

Variable viral kinetics remain a practical challenge because the timing and duration of detectable viral RNA may differ according to viral lineage, vaccination history, previous infection, immune status, and sampling strategy. In practice, test interpretation should therefore remain anchored to symptom timing, exposure history, specimen quality, and anatomical sampling site.

For NGS, specimen suitability remains a separate practical constraint because successful genome recovery depends on viral RNA quantity and quality. Ct-based specimen selection should therefore follow locally validated, assay-specific criteria rather than a universal threshold.

Sequencing programmes should balance technical specimen suitability with the intended sequencing objective, while recognizing that exclusive selection of technically favourable specimens may introduce surveillance bias.

### 7.3. Interpretation of Positive RT-PCR After Vaccination

Viral culture studies indicate that recovery of replication-competent virus is more likely earlier in infection and when viral RNA burden is greater, although this relationship is not absolute [51,52,53,54]. These findings reinforce the need to interpret RT-PCR positivity in a clinical context.

When the clinical significance of a positive result remains uncertain, repeat or alternative testing may be considered according to the clinical context; sequencing is reserved for selected questions such as suspected reinfection, persistent infection, or outbreak investigation.

Prolonged RNA detection should be distinguished from persistent viral replication, which requires additional longitudinal clinical, virological, or genomic evidence.

### 7.4. Assay Performance and Viral Evolution

Viral evolution remains a practical diagnostic challenge because mutations in primer- or probe-binding regions may alter the performance of individual RT-PCR targets [55,56]. Multi-target assays reduce, but do not eliminate, this risk.

Spike-gene target failure in selected multiplex assays illustrated both the potential impact of viral evolution on assay performance and the possible epidemiological value of target-failure patterns [8].

Diagnostic laboratories should use appropriate quality-assurance procedures and monitor target conservation as viral lineages evolve. Genomic and in silico surveillance may help identify mutations potentially affecting assay performance [56,57].

Predicted primer or probe mismatches should be confirmed by analytical or clinical validation before clinically meaningful assay impairment is inferred.

Assay performance should therefore be regarded as dynamic and periodically reassessed as viral lineages change. Manufacturers and laboratories should monitor target conservation, investigate unusual target-failure patterns, document software or assay updates, and communicate clinically relevant performance changes promptly.

### 7.5. Equity, Access, and Implementation Capacity

Unequal access to advanced molecular testing, especially NGS, remains a major diagnostic and surveillance challenge. RT-PCR capacity expanded substantially during the pandemic, but access to high-quality sequencing remains uneven among countries, regions, and healthcare systems. NGS requires sequencing platforms, specialized reagents, trained personnel, quality-control systems, bioinformatic expertise, data-storage capacity, and integration with epidemiological reporting. These requirements create substantial barriers in resource-constrained settings.

Global analyses have demonstrated marked disparities in SARS-CoV-2 genomic surveillance. Chen et al. described substantial heterogeneity in sequencing capacity and data sharing among countries [36]. Brito et al. similarly reported major differences between high-income and low- and middle-income countries in the proportion of cases sequenced and the timeliness of genome submission [58]. Such disparities create surveillance blind spots and may delay recognition of emerging lineages.

Sequence volume alone should not be considered equivalent to surveillance quality. A country may generate many sequences but still obtain a biased representation of viral circulation if sampling is concentrated in selected hospitals, cities, travellers, severe cases, or low-Ct specimens. Representative sampling, metadata completeness, turnaround time, and integration with epidemiological systems are also essential.

Sustainable implementation requires trained personnel, a stable reagent supply, quality assurance, bioinformatic infrastructure, secure data systems, and timely integration of genomic findings into public-health workflows. In resource-constrained settings, sentinel representative sampling, event-triggered sequencing, regional sequencing hubs, and shared bioinformatic resources may provide feasible alternatives to universal sequencing [47,48].

## 8. Opportunities for Integrated Diagnostic Strategies

### 8.1. Objective-Driven Sequencing After Positive RT-PCR

An integrated diagnostic strategy can combine the speed of RT-PCR with the genomic resolution of NGS. RT-PCR remains the first-line method for confirming acute SARS-CoV-2 infection, whereas selected RT-PCR-positive specimens may subsequently be prioritized for sequencing according to predefined surveillance, clinical, or epidemiological objectives.

The term “reflex sequencing” may imply automatic sequencing according to a single laboratory rule. In practice, sequencing selection should be objective-driven and should consider both the reason for sequencing and the technical suitability of the specimen.

Sequencing after a positive RT-PCR result should not be automatic for every specimen. The decision should consider whether the requested genomic information is expected to contribute to representative surveillance, outbreak investigation, suspected reinfection, longitudinal assessment of suspected persistent infection, evaluation of an unusual treatment-associated event, or investigation of an unexpected diagnostic pattern.

Technical suitability should be evaluated separately from clinical or public-health priority. Ct value, RNA quantity, specimen integrity, storage conditions, sample availability, and the sequencing protocol may affect genome recovery, but no single universal Ct cutoff should determine eligibility. Locally validated, assay-specific criteria are required.

This integrated approach preserves the efficiency of routine diagnosis while directing sequencing toward questions with a predefined downstream use. Before NGS is requested, the anticipated action should be specified, such as outbreak-control assessment, comparison with a previous infection, longitudinal genomic monitoring, surveillance reporting, or investigation of an assay target failure.

### 8.2. Operational Sequencing Priorities

Targeted sequencing criteria should be designed to maximize clinical and public-health value without compromising representative surveillance. A balanced sequencing programme should combine representative sampling with event-triggered, outbreak-related, and longitudinal sequencing [11,26,47].

Representative sampling is required to estimate circulating lineage distributions without systematically overrepresenting severe cases, high-risk patients, or technically favourable specimens. Targeted sequencing is appropriate for predefined unusual clinical or epidemiological events, including unexpected clusters, suspected reinfection, unusual diagnostic target failures, and selected treatment-associated events.

Rapid sequencing may be prioritized during active outbreaks in hospitals, long-term care facilities, or other congregate settings when the result can still influence cohorting, contact investigation, ward management, or infection-prevention interventions. Rapid implementation of sequencing has demonstrated potential value in investigations of healthcare-associated COVID-19 [43].

Longitudinal sequencing may be appropriate in selected immunocompromised patients when persistent infection with ongoing viral replication is clinically or virologically suspected. Prolonged RT-PCR positivity alone is insufficient. Supporting features may include persistent or recurrent symptoms, serial changes in viral RNA burden, repeated recovery of viable virus where testing is available, or progressive genomic changes across sequential specimens [35,40].

Suspected reinfection may justify sequencing when clinically and epidemiologically distinct infection episodes are appropriately separated and comparison with an earlier specimen is possible. Interpretation should consider the interval between episodes, intervening test results, new exposure or symptoms, lineage differences, genomic distance, and the possibility of persistent infection [41].

Treatment-associated sequencing should be reserved for clearly defined events after targeted antiviral or monoclonal-antibody exposure when viral evolution or reduced susceptibility is suspected. Interpretation should consider treatment timing, validated resistance data, and whether the mutation may have been present before therapy [42].

Severe post-vaccination disease may warrant sequencing when it forms part of an unusual or epidemiologically informative pattern rather than as an isolated indication.

Table 2 summarizes the principal indications for SARS-CoV-2 sequencing and the clinical, epidemiological, and virological findings that may support sequencing in each context.

### 8.3. Combining Molecular, Clinical, Vaccination, and Epidemiological Data

Integrated interpretation requires molecular findings to be considered together with relevant clinical and epidemiological data. RT-PCR confirms detectable SARS-CoV-2 RNA, Ct values may provide assay-specific contextual information, and NGS can provide lineage and mutation-profile information; each result must be interpreted according to its analytical and clinical limitations.

Symptoms, timing of exposure, vaccination status, time since the most recent dose, booster history, vaccine platform, immune status, comorbidities, previous infection, specimen type, and the likely setting of transmission all influence interpretation.

In outbreak investigations, genomic findings should be integrated with symptom-onset dates, sampling dates, contact patterns, patient and staff movement, ward exposure, and background community diversity [33,43].

Integrated interpretation requires collaboration among clinicians, microbiologists, infection-control teams, epidemiologists, public-health authorities, and bioinformaticians. Reports should distinguish directly observed genomic findings from contextual clinical or epidemiological interpretations.

Vaccination information should also be recorded with sufficient detail. A binary “vaccinated/unvaccinated” variable is often inadequate because the interpretation may depend on vaccine type, number of doses, date of the most recent dose, updated formulation, previous infection, and immune status.

### 8.4. Development of Rapid and Standardized Sequencing Workflows

Rapid sequencing workflows are an important opportunity for improving the clinical and public-health use of NGS. Portable sequencing platforms, optimized amplicon protocols, automated library preparation, and faster bioinformatic pipelines can reduce turnaround time and make genomic data more actionable. The ARTIC protocol and associated primer schemes and bioinformatic resources contributed substantially to the rapid implementation of SARS-CoV-2 genome sequencing in multiple laboratories [27]. Rapid sequencing has also been used for the prospective investigation of healthcare-associated COVID-19 [43].

Faster workflows may allow sequencing results to influence outbreak control while transmission is ongoing, particularly when genomic findings can distinguish probable institutional clusters from multiple introductions.

Speed alone is not sufficient. Rapid workflows must maintain adequate genome completeness, read depth, contamination control, consensus accuracy, reproducibility, and version-controlled bioinformatic analysis. Inaccurate or incomplete genomic results generated rapidly may be less useful than slower but reliable results.

Standardized workflows should document specimen-acceptance criteria, controls, minimum coverage and depth requirements, masking rules, contamination assessment, lineage-assignment software and versions, and reporting pathways.

Tools such as Nextstrain and Nextclade can support genomic visualization, clade assignment, mutation analysis, and sequence-quality assessment [59,60]. Their outputs depend on sequence quality, reference data, software versions, and expert interpretation.

Rapid sequencing provides the greatest operational value when it is embedded within a decision pathway. Laboratories and public-health teams should define who receives the result, how quickly it is reviewed, what action may follow, and how uncertainty is communicated. Without such integration, reduced sequencing time may not translate into faster or more effective intervention.

**Figure 2:**
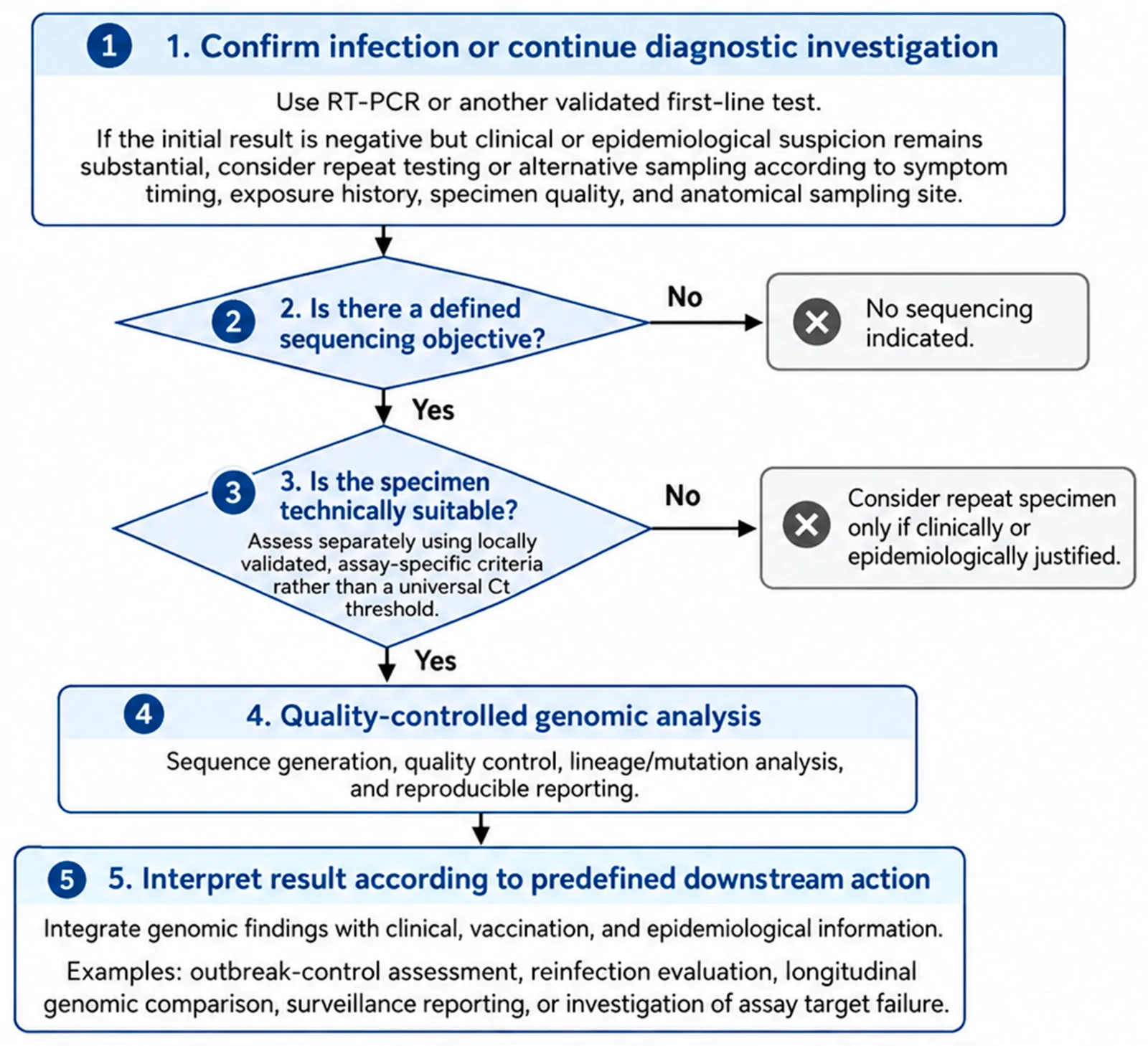
Complementary roles of RT-PCR and next-generation sequencing in post-vaccination SARS-CoV-2 infection.

Figure 2 illustrates the complementary roles of RT-PCR and NGS in post-vaccination SARS-CoV-2 infection.

## 9. Future Perspectives

### 9.1. Point-of-Care and Emerging Molecular Technologies

Future diagnostic systems may increasingly combine rapid point-of-care detection with limited mutation screening. Point-of-care molecular tests can shorten the interval between specimen collection and result reporting, which may be particularly useful in emergency departments, outpatient clinics, long-term care facilities, and geographically remote settings. In the post-vaccination era, such platforms may facilitate timely identification of SARS-CoV-2 infection and support clinical and infection-control assessment.

The diagnostic performance of point-of-care systems varies according to the analytical technology, specimen type, target population, and reference method. Rapid molecular assays generally provide greater sensitivity than antigen-based tests but may require more complex instrumentation and incur higher costs. Their clinical value depends not only on analytical accuracy but also on whether results are available early enough to influence patient placement, support the assessment of treatment eligibility, or inform outbreak-control measures [61].

Recent sample-to-answer microfluidic systems integrate nucleic-acid extraction, amplification, and result interpretation within portable platforms, potentially reducing turnaround time and manual laboratory requirements [62]. Digital PCR may also provide highly sensitive and reproducible nucleic-acid quantification through sample partitioning, although cost, workflow complexity, and limited decentralized availability currently restrict routine point-of-care use.

Another potential direction is the development of assays that detect SARS-CoV-2 while simultaneously screening for a limited number of clinically or epidemiologically relevant mutations. Such systems would not replace NGS because they cannot provide broad genomic characterization or reliably identify unexpected or newly emerging genomic patterns. They may nevertheless serve as rapid triage tools for identifying specimens that warrant further genomic investigation.

CRISPR-based diagnostic systems represent a programmable approach to nucleic-acid detection and may enable rapid mutation-specific testing. Potential advantages include adaptable target recognition, compatibility with isothermal amplification, and integration into portable platforms. However, routine implementation remains constrained by specimen preparation, reagent stability, multiplexing capacity, contamination control, automation, cost, and the need for prospective clinical validation [63].

Mutation-targeted point-of-care assays are also vulnerable to viral evolution because panels designed around currently circulating mutations may lose analytical or epidemiological relevance as lineages change. Their use would therefore require continued genomic surveillance and periodic target updating.

Point-of-care technologies should ultimately be incorporated into broader diagnostic pathways rather than regarded as stand-alone solutions. Discordant or clinically unexpected findings may require confirmatory testing, and specimens selected for genomic investigation must still meet the technical requirements of the sequencing workflow.

### 9.2. Real-Time Genomic Surveillance

Real-time genomic surveillance can strengthen public-health response by integrating genomic, temporal, geographical, and epidemiological information. Platforms such as Nextstrain have demonstrated the value of combining these data to track pathogen evolution and spread [59]. For SARS-CoV-2, timely surveillance can support early recognition of expanding lineages, lineage replacement, and unusual epidemiological patterns.

Operational timeliness depends on the complete surveillance pathway rather than sequencing speed alone. Specimen transport, sequencing, quality control, metadata completion, data submission, analysis, interpretation, and communication to decision-makers all influence whether genomic information becomes available while it remains actionable.

In the post-vaccination setting, surveillance systems should integrate genomic findings with relevant information on vaccination history, previous infection, reinfection, disease severity, treatment exposure, and immune status whenever feasible. A simple vaccinated/unvaccinated classification is often insufficient; more informative surveillance may require vaccine platform, number of doses, date and formulation of the most recent dose, previous infection, and relevant host characteristics, subject to appropriate privacy and data-governance requirements.

Representative and targeted sequencing serve different but complementary purposes. Representative sampling is required to estimate circulating lineage frequencies, whereas targeted sequencing can investigate unusual clinical or epidemiological events. Real-time surveillance systems should incorporate both approaches while analyzing them separately to avoid biased population-level estimates.

Automated dashboards and phylogenetic visualization tools can improve situational awareness, but their outputs remain dependent on sampling density, geographical coverage, metadata quality, and background viral diversity. Computational signals should therefore be used to prioritize investigation and interpretation within an appropriate epidemiological framework.

### 9.3. Advanced Bioinformatics and Machine Learning

Quality-assured bioinformatics is central to genomic surveillance. Computational workflows support sequence-quality assessment, consensus generation, lineage assignment, mutation annotation, phylogenetic analysis, and detection of potential genomic clusters. As sequence volumes increase, standardized and reproducible computational pipelines become increasingly important.

Platforms such as Pangolin and Nextclade support SARS-CoV-2 classification and sequence interpretation [31,60]. Pangolin assigns sequences within the dynamic Pango lineage system, whereas Nextclade supports clade assignment, mutation calling, and sequence-quality assessment. These tools use defined computational algorithms and reference datasets and should not automatically be described as artificial-intelligence applications.

Machine-learning approaches may have future value for analyzing large genomic and epidemiological datasets, including prioritization of unusual genomic patterns or lineages for further investigation. However, such applications remain dependent on representative training data, clearly defined outcomes, external validation, and adaptation to changing epidemiological conditions. Sampling bias, incomplete metadata, delayed sequence submission, and temporal changes in testing practices can substantially affect model performance.

Accordingly, operational genomic surveillance should prioritize validated bioinformatic pipelines, version control, transparent reporting, reproducible quality criteria, and expert interpretation. Machine-learning outputs should not be used for clinical or public-health decision-making without appropriate validation and assessment of uncertainty.

### 9.4. Broader Respiratory-Virus Diagnostics and Surveillance

The laboratory and genomic infrastructure developed for SARS-CoV-2 has potential value beyond COVID-19. Sequencing platforms, bioinformatic networks, quality-assurance systems, and data-sharing mechanisms established during the pandemic may be adapted to broader respiratory-virus surveillance, including influenza viruses, respiratory syncytial virus, and emerging respiratory pathogens.

This transition is clinically relevant because respiratory symptoms caused by SARS-CoV-2, influenza, respiratory syncytial virus, and other pathogens frequently overlap. During periods of concurrent circulation, multiplex molecular assays may improve diagnostic efficiency by detecting and differentiating several respiratory pathogens within a single workflow [64].

A broader surveillance model could combine multiplex molecular testing with representative or targeted sequencing of selected respiratory viruses. Such systems may support monitoring of antigenic drift, antiviral-resistance mutations, vaccine mismatch, changing seasonality, and outbreak spread. However, sequencing and interpretation frameworks must account for differences among viruses in genome structure, evolutionary rate, seasonality, sampling requirements, nomenclature, and the clinical significance of genomic changes.

Wastewater surveillance may provide an additional population-level signal and has been used to monitor SARS-CoV-2, influenza viruses, and respiratory syncytial virus concurrently [65]. Environmental surveillance complements clinical testing but does not provide direct patient-level information on disease severity, vaccination history, or individual transmission events.

The longer-term legacy of SARS-CoV-2 genomic surveillance may therefore be the development of more flexible and resilient systems for respiratory-threat detection. Sustainable preparedness will require trained personnel, interoperable laboratory and data systems, stable infrastructure, shared quality standards, and mechanisms for rapidly adapting diagnostic and sequencing priorities when new respiratory threats emerge.

## 10. Limitations of the Underlying Evidence and of the Present Review

### 10.1. Limitations of the Underlying Evidence

The evidence concerning RT-PCR and NGS in post-vaccination SARS-CoV-2 infection is heterogeneous. Studies differ in design, population characteristics, vaccination and booster history, previous infection, circulating lineage, specimen type, sampling strategy, and diagnostic or sequencing workflow. Findings from one epidemiological period may therefore not be directly applicable to another because viral kinetics, population immunity, antigenicity, and testing practices continue to change.

Vaccination histories have also become increasingly complex. Early studies predominantly evaluated infection after completion of a primary vaccination series, whereas later populations include multiple booster doses, updated vaccine formulations, previous infection, and hybrid immunity. Many studies report vaccination using broad categories without adequately documenting vaccine product, number of doses, timing of the most recent dose, or previous infection, limiting comparability and increasing the potential for residual confounding.

The literature includes substantial numbers of retrospective observational studies, outbreak investigations, convenience samples, and selected clinical cohorts. Sample sizes are often limited for specific subgroups, and definitions of post-vaccination infection, reinfection, persistent infection, treatment-associated events, severe disease, and sequencing success are not uniform. These differences limit direct comparison across studies and constrain quantitative synthesis.

Sequencing datasets are additionally affected by selection bias. Severe cases, institutional outbreaks, immunocompromised patients, travellers, and specimens with greater viral RNA abundance may be preferentially sequenced, whereas mild community infections and technically difficult specimens may be underrepresented. Such selection can distort apparent associations among viral lineage, post-vaccination infection, disease severity, and vaccine effectiveness. Targeted sequencing is valuable for investigating unusual events but should therefore be distinguished from representative sampling intended to estimate population lineage distributions.

Ct values represent another major source of heterogeneity. They vary according to assay platform, extraction method, amplification target, specimen type, collection quality, and laboratory procedures and are not directly interchangeable across assays or laboratories [25]. Studies that use Ct values as uncalibrated proxies for viral load or rely on single-time-point measurements may therefore provide limited information on viral kinetics, viable-virus shedding, or transmission risk.

Genomic workflows likewise differ in sequencing platform, enrichment strategy, primer scheme, genome-completeness threshold, read depth, masking criteria, consensus calling, and lineage-assignment software. Incomplete methodological reporting reduces reproducibility and may obscure important technical sources of heterogeneity. Routine consensus sequencing also has limited ability to characterize low-frequency within-host variants unless appropriately validated deep-sequencing approaches are used.

Geographical representation remains uneven because sequencing capacity, bioinformatic infrastructure, and availability of linked clinical metadata differ substantially across countries and regions [36,60]. This imbalance may influence estimates of lineage emergence and circulation and limits the generalizability of findings from highly sequenced populations.

Finally, the evidence base is vulnerable to rapid obsolescence. Conclusions derived from a particular vaccine formulation, viral lineage, therapeutic agent, diagnostic platform, or public-health policy may become less applicable as viral evolution and clinical practice change.

### 10.2. Limitations of the Present Review

The present article is a narrative review informed by a targeted literature search and does not constitute a systematic review or meta-analysis. No formal risk-of-bias instrument or certainty-of-evidence framework was applied. Study selection and interpretation therefore involve narrative judgement, and the review may not include every relevant publication.

The substantial heterogeneity of study populations, vaccination histories, diagnostic assays, sequencing workflows, circulating lineages, and reported outcomes precluded formal quantitative synthesis. Numerical estimates of diagnostic performance, sequencing success, turnaround time, or cost should therefore be interpreted as context-dependent rather than universally applicable.

The review focuses principally on RT-PCR and NGS. Rapid antigen tests, digital PCR, isothermal amplification, CRISPR-based diagnostics, multiplex respiratory assays, and other emerging technologies are discussed only where they provide relevant context and are not reviewed comprehensively.

The rapidly evolving nature of SARS-CoV-2 constitutes an additional limitation. New viral lineages, vaccine formulations, diagnostic technologies, therapeutic-resistance patterns, and surveillance policies may emerge after the final literature search. The conceptual and operational framework presented here should therefore be periodically reassessed in relation to current epidemiology, public-health guidance, and locally validated laboratory performance.

## 11. Conclusions

RT-PCR and NGS have complementary roles in the evaluation of post-vaccination SARS-CoV-2 infection. RT-PCR remains the principal first-line method for rapid confirmation of acute infection because it is widely accessible, comparatively rapid, and clinically actionable. Its analytical performance is high in validated assays, whereas clinical sensitivity depends on specimen type, sampling quality, anatomical site, timing, and viral kinetics. Ct values are assay-specific and should be interpreted only as contextual measures of detectable viral RNA rather than as standardized indicators of infectivity, disease severity, or sequencing eligibility.

NGS provides broader genomic characterization, including lineage assignment, mutation detection, longitudinal genomic comparison, outbreak investigation, and surveillance of viral evolution. Its principal value lies in selected patient-specific investigations and population-level public-health surveillance rather than routine confirmation of acute infection.

Sequencing may be particularly informative in suspected reinfection, active institutional outbreaks, unusual diagnostic target-failure patterns, selected treatment-associated events, and longitudinal assessment of immunocompromised patients with suspected persistent infection. These indications should be supported by relevant clinical, epidemiological, virological, or longitudinal evidence and should not be based solely on vaccination status, disease severity, prolonged RNA detection, or Ct values.

Genomic findings require interpretation within an appropriate clinical and epidemiological framework. Lineage assignment, mutation detection, and genomic relatedness may strengthen specific hypotheses, but their clinical or public-health significance depends on complementary epidemiological, immunological, functional, and, where relevant, therapeutic evidence.

The most effective strategy is therefore an objective-driven integrated model in which RT-PCR or another validated first-line test confirms infection and sequencing is considered only when a defined clinical, epidemiological, or surveillance objective exists. Technical specimen suitability should be assessed separately using locally validated criteria. Representative surveillance should be maintained to estimate population lineage distributions, whereas targeted and longitudinal sequencing should address unusual events and selected patient-level questions.

Each sequencing request should also be linked to a predefined downstream use, such as outbreak-control assessment, reinfection evaluation, longitudinal genomic comparison, assay-performance investigation, or surveillance reporting. This approach preserves the efficiency of routine diagnosis while directing genomic resources toward questions for which sequencing is most likely to provide meaningful incremental value.

Continued SARS-CoV-2 evolution will require sustained integration of molecular diagnosis, genomic surveillance, and epidemiological interpretation. The laboratory infrastructure, bioinformatic capacity, quality-assurance systems, and data-sharing networks developed during the COVID-19 pandemic may also support broader respiratory-virus surveillance. Sustainable implementation will depend on representative sampling, quality-assured and version-controlled workflows, timely data sharing, trained personnel, and continuous linkage of molecular findings with clinical and epidemiological information.

## Figures and Tables

**Table 1:** Complementary roles and key characteristics of RT-PCR and NGS in post-vaccination SARS-CoV-2 infection.

Characteristic	RT-PCR	NGS
Primary purpose	Confirmation of detectable SARS-CoV-2 RNA	Genomic characterization
Primary level of utility	Individual diagnosis	Patient-specific investigation and population surveillance
Turnaround time	Usually hours	Hours in selected rapid workflows to several days
Specimen requirements	High sensitivity with validated assays; dependent on sampling and timing	Requires sufficient RNA quantity/quality and adequate genome coverage
Ct values	Assay-specific contextual information	May assist specimen selection; no universal sequencing cutoff
Genomic information	Limited to predefined targets or mutations	Broader mutation detection and lineage assignment
Unexpected genomic changes	Generally not detected	Detectable within adequately sequenced regions
Immediate clinical utility	High	Usually limited and objective-dependent
Outbreak investigation	Confirms infection	Supports assessment of genomic relatedness
Population surveillance	Limited	Major established application
Cost/infrastructure	Lower; widely available	Higher laboratory and bioinformatic requirements
Principal limitations	Sampling/timing effects; target dependence; assay-specific Ct values	Coverage failure; sequencing bias; contamination; bioinformatic and interpretive limitations

Abbreviations: Ct, cycle threshold; NGS, next-generation sequencing; RT-PCR, reverse-transcription polymerase chain reaction. Ct values are assay-dependent and should not be interpreted as standardized quantitative measures or universal sequencing thresholds.

**Table 2:** Operational considerations supporting selected sequencing indications.

Sequencing Indication	Findings That May Support Sequencing
**Suspected reinfection**	Clinically and epidemiologically distinct episodes; appropriate interval between episodes; intervening testing; new exposure or symptoms; availability of paired specimens
**Suspected persistent infection**	Prolonged or recurrent RNA detection plus compatible clinical course, longitudinal viral RNA kinetics, culture evidence where available, or genomic evolution across sequential specimens
**Treatment-associated event**	Defined antiviral or monoclonal-antibody exposure; persistent or recurrent infection; suspected viral evolution or reduced therapeutic susceptibility
**Active institutional outbreak**	Temporally and spatially linked cases for which genomic information may influence infection-control assessment
**Unusual clinical or epidemiological event**	Unexpected cluster, diagnostic target failure, unusual lineage pattern, or other predefined event with potential clinical or public-health consequence
**Representative surveillance**	Systematic sampling designed to estimate circulating lineage distributions without preferential selection based on severity or technical convenience

## Data Availability

No new data were created or analyzed in this study. Data sharing is not applicable to this article.
